# Fossil-informed biogeographic analysis suggests Eurasian regionalization in crown Squamata during the early Jurassic

**DOI:** 10.7717/peerj.17277

**Published:** 2024-04-30

**Authors:** Ian V. Wilenzik, Benjamin B. Barger, R. Alexander Pyron

**Affiliations:** Department of Biology, George Washington University, Washington D.C., United States of America

**Keywords:** Biogeography, Squamata, Laurasia, Gondwana, Fossils, DEC, Jurassic, Lizards, Snakes

## Abstract

**Background:**

Squamata (lizards, snakes, and amphisbaenians) is a Triassic lineage with an extensive and complex biogeographic history, yet no large-scale study has reconstructed the ancestral range of early squamate lineages. The fossil record indicates a broadly Pangaean distribution by the end- Cretaceous, though many lineages (e.g., Paramacellodidae, Mosasauria, Polyglyphanodontia) subsequently went extinct. Thus, the origin and occupancy of extant radiations is unclear and may have been localized within Pangaea to specific plates, with potential regionalization to distinct Laurasian and Gondwanan landmasses during the Mesozoic in some groups.

**Methods:**

We used recent tectonic models to code extant and fossil squamate distributions occurring on nine discrete plates for 9,755 species, with Jurassic and Cretaceous fossil constraints from three extinct lineages. We modeled ancestral ranges for crown Squamata from an extant-only molecular phylogeny using a suite of biogeographic models accommodating different evolutionary processes and fossil-based node constraints from known Jurassic and Cretaceous localities. We hypothesized that the best-fit models would not support a full Pangaean distribution (i.e., including all areas) for the origin of crown Squamata, but would instead show regionalization to specific areas within the fragmenting supercontinent, likely in the Northern Hemisphere where most early squamate fossils have been found.

**Results:**

Incorporating fossil data reconstructs a localized origin within Pangaea, with early regionalization of extant lineages to Eurasia and Laurasia, while Gondwanan regionalization did not occur until the middle Cretaceous for Alethinophidia, Scolecophidia, and some crown Gekkotan lineages. While the Mesozoic history of extant squamate biogeography can be summarized as a Eurasian origin with dispersal out of Laurasia into Gondwana, their Cenozoic history is complex with multiple events (including secondary and tertiary recolonizations) in several directions. As noted by previous authors, squamates have likely utilized over-land range expansion, land-bridge colonization, and trans-oceanic dispersal. Tropical Gondwana and Eurasia hold more ancient lineages than the Holarctic (Rhineuridae being a major exception), and some asymmetries in colonization (e.g., to North America from Eurasia during the Cenozoic through Beringia) deserve additional study. Future studies that incorporate fossil branches, rather than as node constraints, into the reconstruction can be used to explore this history further.

## Introduction

The oldest stem-group squamates (lizards, snakes, and amphisbaenians) date back ∼240 million years ago to the Middle Triassic Period of Europe (Simoes et al., 2018). After an early radiation dated to the middle Jurassic in Eurasia (Rage, 2013), crown squamates underwent worldwide diversification, leading to one of the most diverse groups of terrestrial vertebrates with over 11,000 extant species (Uetz, Freed & Hošek, 1995) and over 1,000 extinct species described (Caldwell, 2005). Today, squamates have a nearly global distribution, excluding Antarctica (Vitt & Caldwell, 2014). Numerous studies have examined the recent biogeographic history of lineages such as amphisbaenians, anguimorphs, dibamids, geckos, skinks, and snakes (*e.g.*, Barley et al., 2015; Chen et al., 2017; Fuller, Baverstock & King, 1998; Gamble et al., 2011; Longrich et al., 2015; Noonan & Sites Jr, 2010; Poe et al., 2017; Townsend, Leavitt & Reeder, 2011; Vidal et al., 2010). However, there is a distinct paucity of biogeographic analyses of Squamata as a whole.

Crown Squamata dates to the Jurassic ∼180–190 Ma (Jones et al., 2013; Tonini et al., 2016; Title et al. 2024), around the final breakup of Pangaea (Dietz & Holden, 1970a; Dietz & Holden, 1970b). Many extant lineages (*e.g.*, Pleurodonta, Boidae) show a classical Gondwanan origin (Noonan & Chippindale, 2006a; Noonan & Chippindale, 2006b; Noonan & Sites Jr, 2010), though other lineages exhibit recent trans-oceanic dispersal (Vidal et al., 2008; Townsend, Leavitt & Reeder, 2011; Longrich et al., 2015). Unlike amphibians, a group that shows high endemism in former Laurasian subcontinents including temperate North America and Europe (Duellman, 1999; Pyron, 2014a), few ancient relict squamate lineages share this endemism, with a major exception being Rhineuridae in Florida. Instead, most ancient endemics are restricted to tropical regions such as Amazonia (Aniliidae), Sundaland (Anomochilidae, Lanthanotidae), Madagascar and the Mascarene Islands (Bolyeriidae, Xenotyphlopidae), and Southeast Asia (Shinisauridae). Finally, the most diverse lineages (*e.g.*, Colubridae, Gekkota, Scincidae) are nearly cosmopolitan, suggesting complex patterns of dispersal and vicariance within and among most major continental regions (Carranza & Arnold, 2003; Gamble et al., 2008; Vidal et al., 2008; Chen et al., 2013; Pereira & Schrago, 2017).

There have been several attempts to reconstruct the Squamate Tree of Life to better understand their evolutionary history from a phylogenetic perspective, incorporating species-level sampling and fossil data (see Simões & Pyron, 2021). In contrast, few studies have attempted to study the complex early biogeographic history of squamates (see Evans, 2003), unlike other diverse global radiations like amphibians (Pyron, 2014a) or birds (Field & Hsiang, 2018; Selvatti et al., 2022). No studies have performed a comprehensive estimation of squamate biogeography using a fully sampled phylogeny (*e.g.*, Tonini et al., 2016) and spatial dataset (*e.g.*, Roll et al., 2017) with explicit ancestral range estimation (Matzke, 2014), in part due to difficulty defining spatially homologous regions that are coherent across the timescale of squamate evolution. Recent discoveries such as gekkotans preserved in amber (such as *Cretaceogekko*; Daza et al., 2016) and fossil-tip-based analyses may imply older diversification times for some clades (see Simões & Pyron, 2021) but this is beyond the scope of our study. We therefore utilize a published, dated, species-level phylogeny as the basis for our comparative analyses.

We also emphasize the difference between an “area” and a “range”, with an area being the single biogeographic unit (*i.e.,* the North American plate) and a range being the combination of one or more areas (*i.e.,* an ancestral reconstruction of North America + Eurasia). The delineation of biogeographic areas is a difficult problem with a long history of attempts and without clear solutions in many cases (see Nelson, 1978 for a historical perspective; Morrone, 2018). Some proposed solutions include spatial homology (Escalante, 2017) and network approaches (Vilhena & Antonelli, 2015). Even well-known transition zones such as Sundaland-Oceania have myriad lines (Wallace’s, Weber’s, Lydekker’s, *etc*.) demarcating their boundaries for different taxa in ways that are difficult to integrate (see Simpson, 1977) and reflect differing historical responses to climatic, biotic, and geological forces. Some systems lend themselves well to discrete classifications, such as presence or absence on islands (Ree & Smith, 2008). At global scales, one might choose classifications based on traditionally defined ecoregions (*e.g.*, Pyron, 2014a; Pyron, 2014b), typically delimited from empirical distributions of taxa based on bioregionalization (Kreft & Jetz, 2010). For widely distributed, ancient taxa with distribution patterns driven by paleogeographic processes (*e.g.*, tectonic vicariance), continental or plate-level endemism appear the most appropriate coding scheme (*e.g.*, Bossuyt et al., 2006).

Consequently, compiling distributional data for squamate biogeography presents several hurdles. First, there are many squamate fossils in regions where they are not currently found (Crisp, Trewick & Cook, 2011). Fossil localities throughout the world show extralimital Cenozoic distributions for clades such as Elapidae, Teiidae, Tropidophiidae, and Varanidae in Europe (Szyndlar, Smith & Rage, 2008; Ivanov et al., 2018; Georgalis et al., 2019; Louis & Santiago, 2020), or tropical iguanians from central North America (Conrad, Rieppel & Grande, 2007; Conrad, 2015). In addition, Mesozoic lineages such as Paramacellodidae (Pangaea), Polyglyphanodontidae (North America and Asia), Mosasauroidea (marine environments worldwide), and Madtsoiidae (Gondwana) reveal historical dynamics invisible to analyses of extant taxa (Evans, 2003; Longrich, Bhullar & Gauthier, 2012; Bittencourt et al., 2020), especially for groups more deeply nested within the tree. A growing literature of amber-preserved fossils may expand our understanding of paleobiogeography (Wang & Xing, 2020). An example is *Cretaceogekko*, a proposed gekkotan (Daza et al., 2016) that would shorten the current ghost lineage of Gekkota by about 25 Ma from the previously oldest gekkotan, *Gobekko* (Daza et al., 2016). However, no amber squamates fossils exist before the late Cretaceous (Wang & Xing, 2020) and amber fossils often lack osteological features needed for a phylogenetic analysis, limiting their use. Furthermore, shifting tectonic plates have corresponded with shifting climates and ecosystems (Lomolino, Lomolino & Lomolino, 2010). As a result, historical ecoregions may not be equivalent to their present-day counterparts.

What, then, can we hope to infer accurately from available phylogenetic (Tonini et al., 2016) and spatial (Roll et al., 2017) data? We use fine-scale maps for tectonic plate boundaries (Bird, 2003) to classify squamates into nine major plates involved in Pangaean vicariance to decrease ambiguity associated with delimiting terrestrial ecoregions. While these data were generated in 2003, the definition of continental divisions has remained relatively consistent across recent studies (Seton et al., 2012; Hasterok et al., 2022). We also include node constraints based on fossil occurrences that can be confidently assigned to early squamate lineages (*e.g.*, Evans, 2003). With a suite of model-based inferences, we ask an overarching question: is there a discernable biogeographic signal for ancestral range estimation and endemic regionalization in early Squamata, particularly when fossil geographic occurrences of crown squamates from the Jurassic and early Cretaceous are including as geographic constraints? We find support for this hypothesis regarding a Eurasian origin, and characterize several major patterns in Mesozoic and early Cenozoic squamate biogeography that can be tested in future studies.

## Materials & Methods

### Ranges & areas

**Figure 1:**
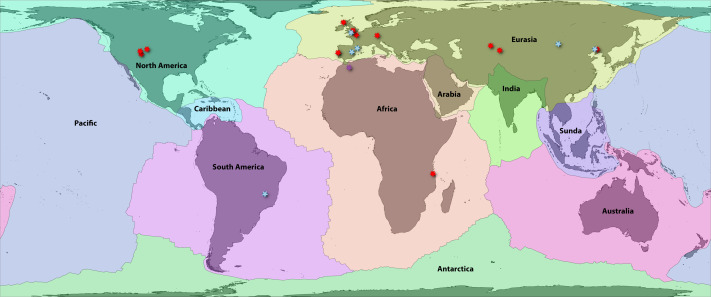
Fossil localities of Jurassic squamates. Map of earth with major plate boundaries adapted from Bird (2003). Red stars indicate the present-day localities of Jurassic squamate fossils (see Table 1), blue represent earliest Cretaceous records (Bittencourt et al., 2020), and purple indicate records across both horizons (Sigogneau-Russell, Monbaron & Russell, 1988; Lasseron et al., 2020).

Bird (2003) provided a high-resolution boundary dataset for 54 major, minor, and microplates. These are highly correlated (and causally linked) with terrestrial zoogeographic regions in many respects (Holt et al., 2013; Ficetola, Mazel & Thuiller, 2017), and by extension, previous global area classifications for groups such as amphibians (Bossuyt et al., 2006; Pyron, 2014a; Vilhena & Antonelli, 2015). First, we aggregated these 54 boundaries to the 8 major plates: Africa, Antarctica, Australia, Eurasia, India, North America, Pacific, and South America. We then separated three minor plates that intersect continental areas with endemic or transitional faunas: Arabia, Caribbean, and Sunda (Fig. 1; Appendix S1). We included these as distinct plates for area coding so that their faunas were not artifactually linked to the adjacent continental plates, which had formed distinct areas prior to the emergence of those three landmasses. Alternatively, Caribbean species occurring on recently emerged landmasses such as the Bahamas would be characterized as “North American,” which does not reflect reality in the Mesozoic.

These 11 plate categories describe most of the major qualitative biogeographic regionalizations (see Lomolino, Lomolino & Lomolino, 2010) and of squamate diversity (see Vitt & Caldwell, 2014), while also reflecting an objective paleogeographic reference. Antarctica contains no extant species and very few (primarily marine) fossils and was therefore omitted from further analyses (Legendre et al., 2020). Additionally, the Pacific plate currently contains substantial landmasses (*e.g.*, Baja California, eastern Melanesia, and southern New Zealand) for which the biogeographic history of their squamate fauna is linked closely to the proximal zoogeographic region (*e.g.*, the Nearctic, Oceania). Therefore, we assigned all Pacific species to their nearest major continental plate. We also opted to lump Madagascar in with Africa for this analysis. Usually, Madagascar is counted as its own plate, as it has a unique tectonic history (*e.g.*, Bossuyt et al., 2006). However, the plate has remained relatively close to Africa since the middle Jurassic, so we argue that treating it as a separate unit is less redundant for our analysis given its exceptional level of squamate endemism, unlike groups such as frogs. This has the added benefit of decreasing the complexity of our models. This resulted in nine major global tectonic ecoregions encompassing nearly all extant, described squamate diversity (Fig. 1; Appendix S1).

To determine species occupancy in the nine regions, we first intersected the 10,064 polygon range shapefiles from the Roll et al. (2017) dataset with the plate boundaries for the 11 major plates, removed Antarctica and reassigned the Pacific species. We inspected these assignments for obvious errors, such as recent human-mediated transplant between continents and a few geometry problems. We then matched these species to the 9,755 taxa in the Tonini et al. (2016) phylogeny, for which 9,569 matched natively and 13 were matched to synonyms, for a total of 9,582 species coded using the Roll et al. (2017) maps. We downloaded range-map shapefiles for a further 64 seasnake taxa (Elapidae: *Aipysurus, Emydocephalus, Ephalophis, Hydrelaps, Hydrophis, Kerilia, Kolpophis, Laticauda, Parahydrophis,* and *Thalassophis*) from the 5 February 2020 update of the IUCN RedList ( http://www.iucn.org/) and classified them similarly. The 109 remaining species were verified manually using the Reptile Database (http://www.reptile-database.org/) or estimated from the literature. We thereby classified all 9,755 species in the Tonini et al. (2016) phylogeny to the nine plates (Appendix S1). Species were limited in their ranges to a maximum of four plates, as almost no species has a range exceeding four. The exceptions were a few species from the marine sea snake genus *Hydrophis*. We constrained those that exceed four plates to the ancestral range of *Hydrophis* (Australia, Eurasia, India, and Sunda) as estimated from the literature (Ukuwela et al., 2017). This was done to reduce the amount of both computational and analytical complexity (see Matzke, 2014).

No biographic coding scenario can account for all possible processes, and some recent Cenozoic patterns may be obscured by our scheme. Examples include the boundary between southeastern Eurasia and Sundaland or between Sundaland and Australasia (Oceania) and the corresponding faunal “lines” discussed by Simpson (1977). Similar criticisms may be leveled at the grouping of most of Central America with Jamaica, Puerto Rico, and the Lesser Antilles on the Caribbean plate, while grouping Cuba and Hispaniola on the North American plate (Fig. 1). The biogeographic history of Caribbean herpetofauna is complex, and this paleogeographical approach accounts for only a portion of recent historical processes (Rosen, 1975; Hedges, 1982; Crother & Guyer, 1996). Regardless, we suggest that this framework is a solid foundation for understanding early squamate biogeography. We anticipate future researchers will refine and revise these classifications using other quantitative methods such as network-based bioregionalization (Vilhena & Antonelli, 2015), incorporating spatial occurrence data and paleogeography to estimate finer-scale processes of recent dispersal and vicariance. Ultimately, we recorded 2,104 species in Africa, 344 in Arabia, 1,529 in Australia, 951 in the Caribbean, 1,202 in Eurasia, 702 in India, 1,173 in North America, 1,953 in South America, and 1,326 in Sundaland. This sums to 1,898 species in landmasses from Laurasian North America and 2,271 in Laurasian Eurasia, and 6,381 in Gondwanan continents and subcontinents.

**Figure 2:**
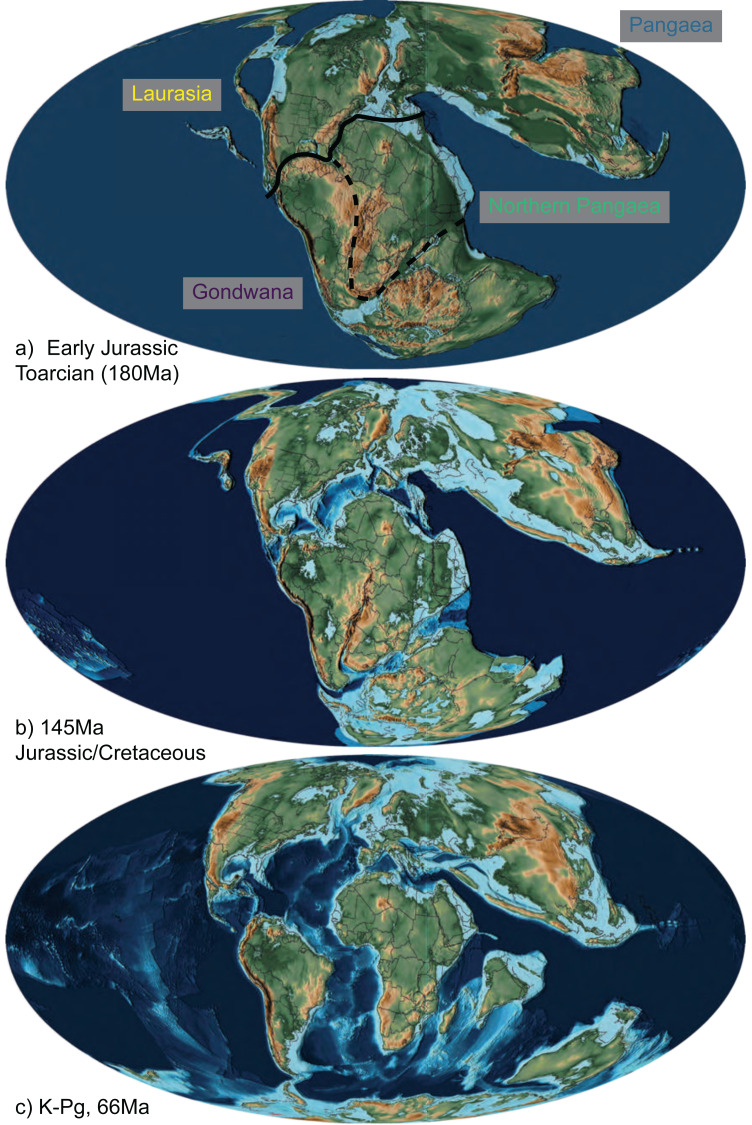
The abbreviated geographic regions in the Mesozoic. Map of Jurassic and Cretaceous paleocontinents from Scotese (2016) drawn at the (A) Early Jurassic (Toarcian, 180Ma), with the three areas of Laurasia (yellow) and Gondwana (purple), Northern Pangaea (green; the boundary of which is Laurasia plus the area designated by the dashed line), and Pangaea (blue) indicated with their transition boundaries; (B) Jurassic/Cretaceous boundary (145 Ma), at which time fossil squamates are known from all three combined areas (Fig. 1; Table 1; Evans, 2003); and (C) K-Pg boundary (66 Ma), after which we see a significant decrease in relative dispersal probabilities between areas (Table 2; Fig. 4).

Note that with nine areas and four allowed in a range, there are 256 possible states—too many to be visualized individually. Only 66 of these were occupied by squamates in our estimates. For visualization purposes, we primarily present our results summarized into four major synthetic, *post-hoc* ranges based on the ancestral estimates. These are Gondwana (Australia, Africa, Arabia, India, and South America), Laurasia (Eurasia, Caribbean, North America, and Sunda), Pangaea (any Gondwanan + Laurasian range), and Northern Pangaea (Laurasia + Africa). These areas are descriptive and not meant to be exclusive of each other or restricted to landmasses from which their names originated. Consequently, if a clade is reconstructed to have a “Laurasian” origin, that does not necessarily mean the clade originated during the Jurassic while Laurasia was still a united supercontinent; rather, it means that the lineage originated in an area arising from the paleocontinent (*i.e.,* North America, Eurasia, Sunda, and the Caribbean), even if the lineage postdates fragmentation in the Cenozoic. If an ancestral range reconstructs areas belonging to both Gondwana and Laurasia, then it is considered to have a “Pangaean” distribution. The full 9-area results can be seen in Appendix S1. Therefore, while we collapse many Cenozoic biogeographic patterns into “Gondwana” and “Laurasia” for ease of illustration (Fig. 2), complex patterns among the 9 areas are present, particularly since the K–Pg boundary (Appendix S2).

**Figure 3:**
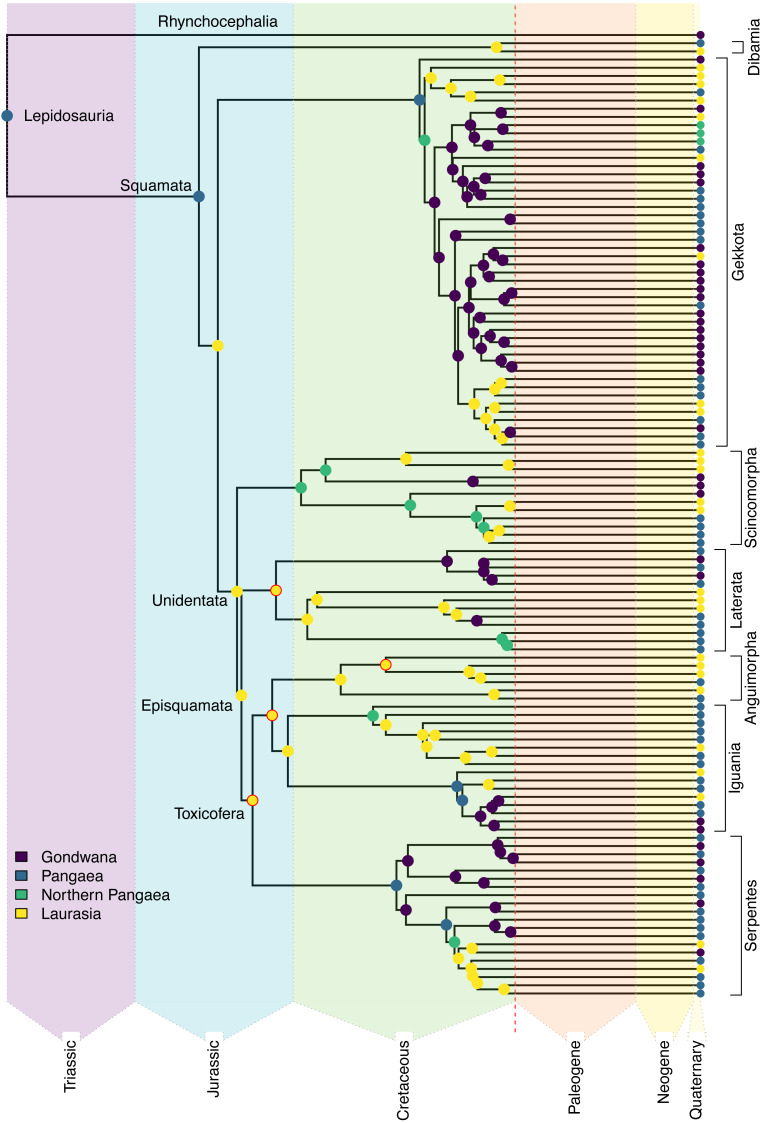
Ancestral state reconstruction of Mesozoic squamates. Reduced representation of the squamate backbone tree (Tonini et al., 2016), showing the best-fit estimates from the DEC+J model (see Appendix S1 for full results and uncertainty) for the geographic origins of early squamate lineages. Named clades of particular interest are discussed in the Results section. The nodes that were constrained are highlighted in red. The color scheme for the major combined areas is consistent throughout the rest of the article.

### Ancestral range estimation

We used the BioGeoBEARS package (Matzke, 2018) in R 3.6.0 to test several candidate models of biogeographic inference incorporating dispersal, extinction, cladogenesis, and founder-event speciation (Matzke, 2012). We tested the commonly used biogeographic models DEC (Ree & Smith, 2008), DIVA (Ronquist, 1997), and BAYAREA (Landis et al., 2013) with and without the addition of the free *‘j’* parameter, which allows for founder-event-speciation as a possible explanation for range expansion during cladogenesis (Matzke, 2014). Despite criticisms surrounding the *‘j’* parameter (Ree & Sanmartín, 2018), recent results demonstrate the validity of this approach (Matzke, 2021) when modeled and interpreted appropriately, as we are mindful to do here. We did not evaluate other possible parameters such as *‘x’* and *‘n’* (dispersal probability as a function of physical or ecological distance; see (Dam & Matzke, 2016)), or trait-based dispersal models (Klaus & Matzke, 2020), as these are less relevant to the questions here and difficult to optimize for a dataset of this size.

Ancestral range estimates typically produce partial support for multiple possible models. For example, an ancestral range of “Africa” may have 70% support, with 25% for “Africa + Arabia,” and 5% for “Arabia” alone for a given node. Consequently, “Africa” as an ancestral area occurs in 95% of the model space for that node. As we had nine geographic areas included in our analysis, we tested differences in total contribution to the ancestral range estimation from each area. A higher total probability means that the area is included across a greater proportion of estimated potential ancestral ranges. Using this method, we calculated the proportions for all nine areas for 11 major nodes (Squamata, Unidentata, Episquamata, Toxicofera, Dibamidae, Gekkota, Scincomorpha, Lacertoidea, Anguimorpha, Iguania, Serpentes; Fig. 3). From this, we can identify geographic areas of outsized importance in early squamate evolution.

Limitations on biogeographical analyses are not usually related to size of the tree used, but rather the number of areas in the model (Matzke, 2012; Landis et al., 2013). We used the 9,755-taxon tree from Tonini et al. (2016), pruned to the 5,415 species which had molecular data. This includes most described, extant genera, over 50% of all known, extant species, and covers the full range of biogeographic diversity in the group. While some of the calibration for the phylogeny may be updated in the future (see Simões & Pyron, 2021; Title et al., 2024), that concern is beyond the scope of our current study. We also considered the use of the backbone topology of 5,415 species with molecular data superior to the use of the fully sampled posterior distribution with imputed species. The latter would have required us to integrate ancestral area estimates across a sample of trees, a procedure of unclear statistical and biological validity (NJ Matzke, pers. comm.). We compared model fit using the likelihood-ratio test along with AICc to estimate relative likelihoods and select the best-fit model from the set of candidate models (Wagenmakers & Farrell, 2004). Future analyses may incorporate correspondence classes and fossil geographic occurrences to qualify area connectivity due to plate tectonics more finely in ancestral range estimation (*e.g.*, Landis, 2017), particularly for trees including extinct or fossil lineages (*e.g.*, Pyron, 2017; Simoes et al., 2018).

### Fossil area constraints

In order to explore the intricate biogeographic history of Squamata, we must first interpret their early fossil record (see Evans, 2003) with respect to recent advances in phylogenetic understanding (Simões & Pyron, 2021). We compiled all verified records of Jurassic and early Cretaceous species from Pangaean landmasses, ranging from the Toarcian to Barremian (Table 1). Recent literature suggests that ancestral range estimation using extant-only datasets may fail to accurately reconstruct ancestral ranges for ancient nodes (Silvestro et al., 2016; Wisniewski, Lloyd & Slater, 2022). To address this issue, we ran models utilizing constraints from geographic occurrences derived from the Jurassic and early Cretaceous fossil record, referred to as “nodal constraints” hereafter. Nodal constraints match fossils of a specific clade to the ancestral lineage for that clade in the phylogeny. Once the fossil taxon has been matched to a node, the node is constrained so that its ancestral range must include the range of which the fossil is located. For instance, if fossil A, located in North America, is used to constrain node 3, then the ancestral range for node 3 must contain “North America” along with any other estimated areas.

**Table 1:** List of Jurassic and Early Cretaceous Fossil Squamates. Fossil squamate species from Jurassic and early Cretaceous localities (see Evans, 2003; Xing et al., 2018 for similar compilations).

**Species**	**Age**	**Locality**	**Depositional environment**	**Source**
*Ardeosaurus brevipes*	Tithonian, Late Jurassic (150.8–145.5 Ma)	Solnhofen Limestone, Germany	Lagoon	Estes (1983)
*Ardeosaurus digitatellus*	Tithonian, Late Jurassic (150.8–145.5 Ma)	Solnhofen Limestone, Germany	Lagoon	Hoffstetter (1964)
*Balnealacerta silvestris*	Bathonian, Middle Jurassic (167.7–164.7 Ma)	Kirtlington Cement Works Quarry, Oxfordshire, United Kingdom	Mixed coastal lake and pond	Evans (1998)
*Bavarisaurus macrodactylus*	Tithonian, Late Jurassic (150.8–145.5 Ma)	Solnhofen Limestone, Germany	Lagoon	Estes (1983)
*Becklesius hoffstetteri*	Kimmeridgian, Late Jurassic (155.7–150.8 Ma)	Guimarota Mine, Leiria, Portugal Alcobaca formation, Portugal	Lagoon	Seiffert (1975)
*Bellairsia gracilis*	Bathonian, Middle Jurassic (167.7–164.7 Ma)	Kirtlington Cement Works Quarry, Oxfordshire, United Kingdom	Mixed coastal lake and pond	Evans (1998)
*Bharatagama rebbanensis*	Toarcian, Early Jurassic (183.0–171.6 Ma)	Kota Formation, Paikasigudem, India	Terrestrial: claystone and sandstone	Evans, Prasad & Manhas (2002)
				But see: Conrad (2018)
*Changetisaurus estesi*	Bathonian, Middle Jurassic (167.7–164.7 Ma)	Balabansai formation, Jalal-Abad, Krygyzstan	Terrestrial: red claystone	Federov & Nessov (1992)
*Diablophis gilmorei*	Kimmeridgian, Late Jurassic (150.8–145.5 Ma)	Morrison Formation, Colorado USA	Fluvial	Evans (1996)
*Dorsetisauridae sp.*	Kimmeridgian, Late Jurassic (155.7–145.5 Ma)	Morrison Formation, Como Bluff, Wyoming	Fluvial	Prothero & Estes (1980)
*Dorsetisaurus purbeckensis*	Kimmeridgian, Late Jurassic (155.7–145.5 Ma) Middle Berriasian, Early Cretaceous (145–140.2 Ma)	Alcobaça Formation, Portugal Lulworth Formation, England, United Kingdom	Lagoon Marine	Hoffstetter (1967)
*Durotrigia triconidens*	Oxfordian, Late Jurassic (161.2–155.7 Ma)	Cordebugle, Lisieux, Basse-Normandie, France	coarse channel fill; concretionary, ferruginous, conglomeratic sandstone	Hoffstetter (1967)
*Eichstaettisaurus schroederi*	Tithonian, Late Jurassic (150.8–145.5 Ma)	Solnhofen Limestone, Germany	Lagoon	Hoffstetter (1964)
*Eophis underwoodi*	Bathonian, Middle Jurassic (167.7–164.7 Ma)	Kirtlington Cement Works Quarry, Oxfordshire, United Kingdom	Mixed coastal lake and pond	Caldwell et al. (2015)
*Eoscincus ornatus*	Tithonian, Late Jurassic (150–145 Ma)	Morrison Formation, Dinosaur National Monument, Utah	Fluvial	Brownstein et al. (2022)
*Hongshanxi xiei*	Oxfordian, Late Jurassic (161.2–155.7 Ma)	Tiaojishan Formation, Guanchaishan, China	Lacustrine	Dong et al. (2019)
*Introrsisaurus pollicidens*	Kimmeridgian, Late Jurassic (155.7–150.8 Ma)	Guimarota Mine, Leiria, Portugal Alcobaca formation, Portugal	Lagoon	Hoffstetter (1967)
*Microteras borealis*	Tithonian, Late Jurassic (150–145 Ma)	Morrison Formation, Dinosaur National Monument, Utah	Fluvial	Brownstein et al. (2022)
*Oxiella tenuis*	Bathonian, Middle Jurassic (167.7–164.7 Ma)	Kirtlington Cement Works Quarry, Oxfordshire, United Kingdom	Mixed coastal lake and pond	Evans (1998)
*Palaeolacerta bavarica*	Tithonian, Late Jurassic (150.8–145.5 Ma)	Solnhofen Limestone, Germany	Lagoon	Estes (1983)
*Parviraptor estesi*	Bathonian, Late Jurassic (167.7–164.7 Ma)	Kirtlington Cement Works Quarry, Oxfordshire, United Kingdom	Mixed coastal lake and pond	Evans (1994)
*Paramacellodus oweni*	Kimmeridgian, Late Jurassic (155.7–145.5 Ma) Kimmeridgian, Late Jurassic (155.7–150.8 Ma) Bathonian, Middle Jurassic (167.7–164.7 Ma)	Morrison Formation, Como Bluff, Wyoming Dinosaur National Monument, Utah Kilmaluag Formation, Scotland	Fluvial Lagoon	Hoffstetter (1967) Evans & Chure (1998) Waldman & Savage (1972)
*Paramacellodidae indet.*	Upper Jurassic (161.2–145.5 Ma)	Tendaguru Formation, Tanzania	Sandstone	Broschinski (1999)
*Portugalophis lignites*	Kimmeridgian, Late Jurassic (155.7–150.8 Ma)	Guimarota Mine, Leiria, Portugal Alcobaca formation, Portugal	Coal swamps	Caldwell et al. (2015)
*Saurillodon marmorensis*	Bathonian, Middle Jurassic (167.7–164.7 Ma)	Kirtlington Cement Works Quarry, Oxfordshire, United Kingdom	Mixed coastal lake and pond	Evans (1998)
*Saurillodon proraformis*	Kimmeridgian, Late Jurassic (155.7–150.8 Ma)	Guimarota Mine, Leiria, Portugal Alcobaca formation, Portugal	Lagoon	Estes (1983)
*Saurillus henkeli*	Kimmeridgian, Late Jurassic (155.7–150.8 Ma)	Guimarota Mine, Leiria, Portugal Alcobaca formation, Portugal	Lagoon	Seiffert (1975)
*Saurillus obtusus*	Kimmeridgian, Late Jurassic (155.7–150.8 Ma)	Guimarota Mine, Leiria, Portugal Alcobaca formation, Portugal	Lagoon	Seiffert (1975)
*Schillerosaurus utahensis*	Kimmeridgian, Late Jurassic (155.7–145.5 Ma)	Morrison Formation, Dinosaur National Monument, Utah	Fluvial	Nydam, Chure & Evans (2013)
*Schoenesmahl dyspepsia*	Tithonian, Late Jurassic (150.8–145.5 Ma)	Solnhofen Limestone, Germany	Lagoon	Conrad (2018)
*Sharovisaurus karatauensis*	Oxfordian, Late Jurassic (161.2–150.8 Ma)	Kerabastau formation, Kazakhastan	Lacustrine	Hecht & Hecht (1984)
*Becklesius cataphractus*	Late Barremian, Early Cretaceous 130–125.5 Ma	Calizas de la Huérguina Formation, Cuenca, Spain	Lacustrine	Richter (1994b)
*Cuencasaurus estesi*	Late Barremian, Early Cretaceous 130–125.5 Ma	Calizas de la Huérguina Formation, Cuenca, Spain	Lacustrine	Richter (1994b)
*Dalinghosaurus longidigitus*	Late Barremian, Early Cretaceous 130–125.5 Ma	Yixian Formation, Liaoning, China	Fluvial	Evans & Wang (2005)
*Hoyalacerta sanzi*	Late Barremian, Early Cretaceous 130–125.5 Ma	Calizas de la Huérguina Formation, Cuenca, Spain	Lacustrine	Evans & Barbadillo (1999)
*Jucaraseps grandipes*	Late Barremian, Early Cretaceous 130–125.5 Ma	Calizas de la Huérguina Formation, Cuenca, Spain	Lacustrine	Bolet & Evans (2012)
*Liushusaurus acanthocaudata*	Late Barremian, Early Cretaceous 130–125.5 Ma	Yixian Formation, Liaoning, China	Lacustrine	Evans & Wang (2010)
*Meyasaurus crusafonti*	Late Barremian, Early Cretaceous 130–125.5 Ma	La Pedrera de Meià, El Montsec, Spain	Lacustrine	Evans & Barbadillo (1996)
*Meyasaurus diazromerali*	Late Barremian, Early Cretaceous 130–125.5 Ma	Calizas de la Huérguina Formation, Cuenca, Spain	Lacustrine	Evans & Barbadillo (1997)
*Meyasaurus faurai*	Late Barremian, Early Cretaceous 130–125.5 Ma	La Pedrera de Meià, El Montsec, Spain	Lacustrine	Evans & Barbadillo (1996)
*Meyasaurus unaensis*	Late Barremian, Early Cretaceous 130–125.5 Ma	Calizas de la Huérguina Formation, Cuenca, Spain	Alluvial	Richter (1994a)
*Norellius nyctisaurops*	Late Barremian, Early Cretaceous 130–125.5 Ma	Öösh Formation, Ovorkhangai, Mongolia	Terrestrial	Conrad & Daza (2015)
*Pseudosaurillus becklesi*	Middle Berriasian 145.5–140.2 Ma	Lulworth Formation, England, UK	Marine	Hoffstetter (1967)
*Purbicella ragei*	Middle Berriasian 145.5–140.2 Ma	Lulworth Formation, England, UK	Marine	Evans, Jones & Matsumoto (2012)
*Scandensia ciervensis*	Late Barremian, Early Cretaceous 130–125.5 Ma	Calizas de la Huérguina Formation, Cuenca, Spain	Lacustrine	Evans & Barbadillo (1998)
*Yabeinosaurus bicupsidens*	Late Barremian, Early Cretaceous 130–125.5 Ma	Yixian Formation, Liaoning, China	Lacustrine	Dong, Wang & Evans (2017)
*Yabeinosaurus robustus*	Late Barremian, Early Cretaceous 130–125.5 Ma	Yixian Formation, Liaoning, China	Lacustrine	Dong, Wang & Evans (2017)
*Yabeinosaurus tenuis*	Late Barremian, Early Cretaceous 130–125.5 Ma	Yixian Formation, Liaoning, China	Lacustrine	Dong, Wang & Evans (2017)

This method hinges on the proper identification of fossils and assumes robust phylogenies, which are not always available. For instance, within Squamata, the lizard *Jeddaherdan aleadonta* was originally thought to be an iguanid from the middle Cretaceous of Africa. However, upon reexamination, morphological evidence suggested that *Jeddaherdan* was from the late Quaternary and was a member of the genus *Uromastyx* (Vullo et al., 2023). To help address this uncertainty, we chose fossil taxa based on three consistent criteria: (1) the taxon must be from the Jurassic Period or early Cretaceous, (2) the taxon must be close in temporal proximity to a branch in the squamate phylogeny occurring in the Jurassic or early Cretaceous, and (3) the taxon must be confidently assigned to the specific clade designated by the node based on an explicit phylogenetic or character-based hypothesis. This excludes earlier, stem-squamates (*i.e., Megachirella*; Simoes et al., 2018), as they can only be assigned to the base of Squamata, negating the purpose of an ancestral range estimation, or to earlier, non-squamate groups (which would be the rhychocephalian *Sphenodon*) which would be outside of the questions possible using molecular phylogenies of Squamata.

Four taxa met these criteria for use as nodal constraints. The first was the early stem snake *Eophis underwoodi*, discovered in the Kirtlington Cement Works Quarry, in Oxfordshire, United Kingdom from the Bathonian epoch (Middle Jurassic, 167.7–164.7 Ma) (Caldwell et al., 2015). While *Eophis* is considered stem-Serpentes (Caldwell et al., 2015), it is temporally closer to the base of Toxicofera. As all stem and crown Serpentes are toxicoferans, we therefore used this fossil occurrence to constrain the Toxicoferan node to include Eurasia. The next was the iguanid *Introrsisaurus pollicidens*, located in the Guimarota Mine and Alcobaca formation, Portugal from the Kimmeridgian epoch (Late Jurassic, 155.7–150.8 Ma; (Hoffstetter, 1967)). We used this to constrain the Iguania + Anguimorpha node to include Eurasia. The third was *Dalinghosaurus longidigitus* (Paleoanguimoprha), found in the Yixian Formation in the early Cretaceous of Liaoning, China (Barremian, 130–125.5 Ma; (Evans & Wang, 2005)). We used this to constrain Paleoanguimorpha branch to include Eurasia. The final fossil was *Meyasaurus diazromerali*, an early member of Laterata located in the Calizas de la Huérguina Formation in Cuenca, Spain (Early Cretaceous, 129.4–126.3 Ma; (Evans & Barbadillo, 1997)). We used this to constrain the Laterata node to the Eurasian plate. A complete list of the fossil history of Squamata in the Jurassic is included in Table 1.

## Results

### Biogeographic history

Based on the fossil-constrained analysis described above, DEC+J received overwhelming support (AIC_wt ≈1; Table 2). We use this model as our best estimate of squamate biogeography. Ancestral-range estimates (see Appendix S1 for full results) show support for a localized Pangaean origin (Africa, Australia, Eurasia, and Sunda) of extant Lepidosauria (Squamata + Rhynchocephalia), which is congruent with Gondwanan Triassic presence of rhynchocephalians (*e.g.*, Bonaparte & Sues, 2006) and Laurasian Triassic presence of squamates (Simoes et al., 2018). Subsequently, the node subtending extant squamates is estimated to have the same ancestral range of Pangaea (Africa, Australia, Eurasia, and Sunda) ∼180 Ma, some 68 million years after divergence from the rhynchocephalians, approximately coinciding with the beginning split between Gondwana and Laurasia in the Early Jurassic (Fig. 3).

**Table 2:** Model selection using AICc. Results from model testing of squamate biogeography via BioGeoBEARS, with the bestsupported model (DEC+ *j*) in bold. The addition of the ‘*j* ’ parameter did not absorb a disproportionate amount of the dispersal estimated in the other models, a caution expressed by some previous authors (Ree & Smith, 2008; see Matzke, 2021).

**Model**	**lnL**	***k***	***d***	***e***	***j***	**AICc**	**Wt**
DEC	−7,319	2	0.0016	1.00E−12	0	14,642	9.00E−10
**DEC+J**	**−7,297**	** 3**	**0.0015**	**1.00E−12**	**0.0008**	**14,601**	**1**
DIVALIKE	−7,885	2	0.0019	1.00E−12	0	15,774	1.40E−255
DIVALIKE+J	−7,819	3	0.0017	1.00E−12	0.0019	15,645	1.90E−227
BAYAREALIKE	−12,365	2	0.01	0.01	0	24,735	0
BAYAREALIKE+J	−12,278	3	0.01	0.01	0.001	24,561	0

The earliest diverging squamate lineage (Dibamia) exhibits a subsequent contraction from Pangaea into Laurasia (North America, Eurasia, and Sundaland) during the long ghost lineage between their ∼180 Ma divergence from the remainder of Squamata and their most recent common ancestor (MRCA) ∼73 Ma, possibly as the final portions of the Sundaland plate had collided with mainland Eurasia (Metcalfe, 1998). We estimate an ancestral range for Gekkota identical to that of Lepidosauria and Squamata (Africa, Australia, Eurasia, and Sunda). Most of the later-diverging lizard lineages in the Jurassic show an entirely Eurasian distribution, including Episquamata, Unidentata, Toxicofera, Laterata, Anguimorpha, and Iguania (see Burbrink et al., 2020 for clade definitions).

In the early Cretaceous, most stem lineages were regionalized to Laurasia, though we estimate a broad ancestral range for Scincomorpha, which shows a northern Pangaean distribution (Africa and Eurasia). Crown Serpentes also evolved in the Cretaceous, and the estimated ancestral range for extant snakes is also Pangaean (Africa, Eurasia, South America, and Sunda). Within Serpentes, we estimate an African origin for Scolecophidia and a South American origin for Alethinophidia, showing the first examples of Gondwanan regionalization in Squamata. In contrast with widespread Laurasian regionalization in the Jurassic and early Cretaceous, Gondwanan endemic lineages of extant taxa are established by the end Cretaceous in Gekkota, Iguania, Laterata, Scincomorpha, and Serpentes. These arose through several different mechanisms: *via* contraction from a broad Pangaean range in Scolecophidia, Alethinophidia, and Iguania; contraction from a Northern Pangaean range in Scincomorpha, some Gekkota, and colubroid snakes; or dispersal from Laurasia in some Gekkota and Laterata (Fig. 3).

**Figure 4:**
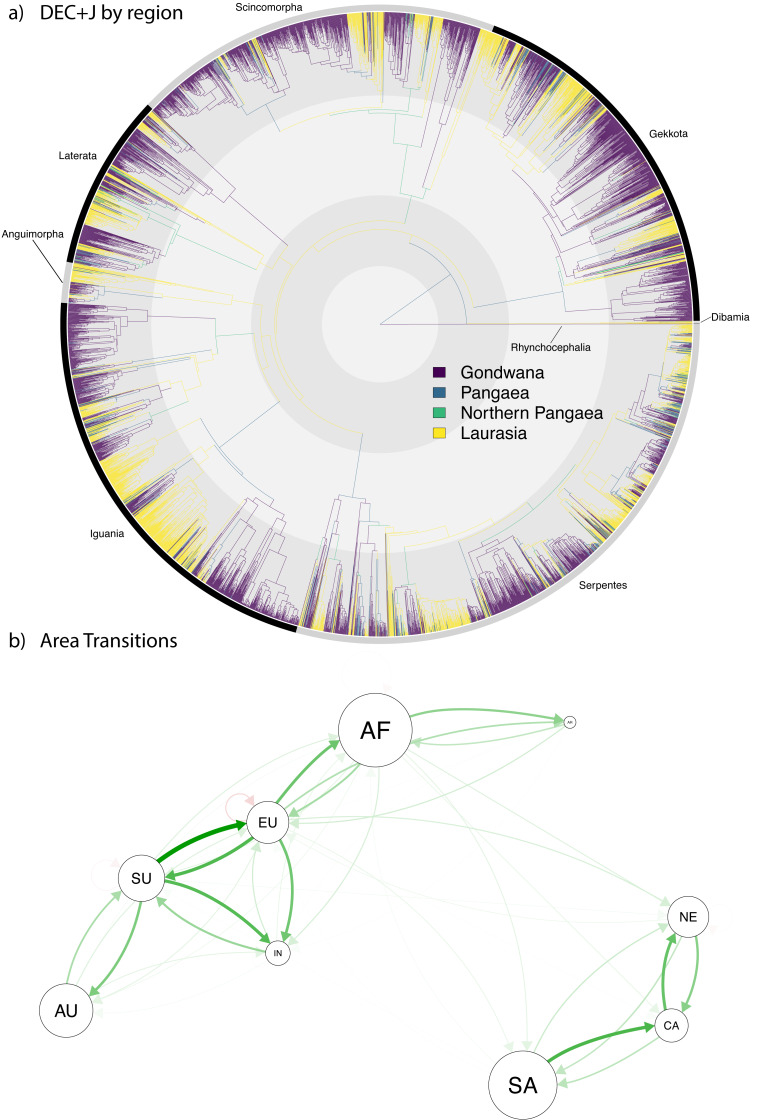
Ancestral range estimation of Squamata into the Cenozoic. A circle tree (Jetz et al., 2012) showing the complete phylogeny of Squamata (Tonini et al., 2016) onto which the ancestral range estimations are mapped (A), which expands the temporal range from the K-Pg in Fig. 3 into the current time. (B) A network analysis of dispersals between the different biogeographic regions. The size of the circles corresponds to extant diversity. The width and density of the arrows correspond to the number of dispersals from one area to another in the direction the arrow is pointing. Red arrows indicate extinction.

In the Cenozoic, we estimate fewer transitions between ancestral areas designated by Laurasia, Gondwana, and northern Pangaea, and observe an increasing frequency of dispersal events within Old World and New World landmasses, with relatively infrequent instances of dispersal between the Old and New Worlds (Fig. 4B). Corresponding to its estimated status as a major locality of origination for Squamata (Table 3), Eurasia continued to act as a source of diversity for squamates throughout the Cenozoic, with numerous dispersals into Africa, India, and Sunda. Similarly, Sunda also acts as a major source area, with frequent dispersals into Eurasia, along with several events into Australia and India. Interestingly, both Eurasia and Sunda also show the highest rates of extirpation (*i.e.,* lineage contraction from an area), with Eurasia having nine lineages contracting from the continental plate and Sunda having two. We also estimated one extinction each in Africa, North America, and India (Fig. 4B).

**Table 3:** Proportional data on ancestral ranges. Proportions for each geographic area in the ancestral range estimations for the major early squamate nodes (note, neither the columns or rows sum to one, as each cell measures the proportion of ancestral range estimations that contain a specific area versus the proportion of ancestral range estimations that do not contain that area). Areas with substantial support ( >0.5) are in bold. For Scincomorpha, a proportion equal to 1 for Eurasia represents a nodal constraint based on fossil data.

Taxon	Africa	Arabia	Australia	Caribbean	Eurasia	India	North America	South America	Sunda
Squamata	**0.50**	0.05	0.48	0.06	**0.89**	0.09	0.45	0.16	**0.61**
Unidentata	0.31	0.01	0.02	0.01	**0.91**	0.02	0.14	0.09	0.10
Episquamata	0.17	0.00	0.00	0.01	**0.88**	0.00	0.08	0.10	0.02
Toxicofera	0.22	0.00	0.00	0.01	**0.87**	0.01	0.04	0.11	0.03
Dibamia	0.04	0.03	0.07	0.03	**0.63**	0.03	**0.65**	0.04	**0.99**
Gekkota	**0.83**	0.01	**0.96**	0.05	**0.78**	0.09	0.39	0.19	0.47
Scincomorpha	**0.86**	0.03	0.04	0.01	**1.00**	0.06	0.26	0.01	0.21
Laterata	0.19	0.01	0.01	0.01	**0.91**	0.01	0.31	**0.50**	0.01
Anguimorpha	0.11	0.03	0.04	0.01	**0.99**	0.03	0.19	0.01	0.18
Iguania	0.26	0.01	0.01	0.08	**0.88**	0.01	0.15	0.16	0.03
Serpentes	**0.91**	0.01	0.04	0.08	**0.58**	0.11	0.07	**0.88**	0.40

Ancestral range estimates for Mesozoic squamate lineages received insignificant contributions from the Arabian, Caribbean, Indian, and South American plates (Table 3). Some of these such as the Caribbean were not land positive in their current form in the Mesozoic, suggesting that these plates were not important areas of origination and diversification of squamate lineages. In contrast, it seems likely that South America and India had diverse endemic squamate faunas that are not represented among extant lineages but may be present undiscovered in the fossil record. For the root node of Squamata, the model shows significant contributions from the African, Eurasian, and Sunda plates as part of the ancestral range. These three continental plates are clearly important for the early evolution of Squamata across the Mesozoic (see Evans, 2003; Simões & Pyron, 2021) and should be an active area of research in the study of ancient squamates, particularly the phylogenetic placement of fossil taxa from those regions.

## Discussion

### Biogeographic models

We estimate overwhelming support for a DEC+J model (Table 2), reflecting strong evidence for long-distance dispersal and colonization (particularly during the Cenozoic), a widely implicated mode of biogeographic range expansion for squamate lineages moving among former Pangaean landmasses currently or previously separated by great distances (Longrich et al., 2015). Recent authors (Ree & Sanmartín, 2018) raised potential issues with biogeographic inference using models (specifically DEC-type models) with founder-event speciation. However, their primary critique lay with cladogenesis parameters, which they claimed are problematic in being modeled probabilistically without respect to time in DEC-type models, an issue they further claim is compounded when additional weight is assigned these events *via* the *‘j’* parameter. We note that our best-fit model (DEC+J) does not seemingly over-estimate the jump parameter in respect to the dispersal, as the dispersal parameter (*d* = 0.0015) was estimated to be an order of magnitude higher than the parameter for the jump dispersal (*j* = 0.008; Table 2). Matzke (2021) showed that these concerns are likely unfounded for most empirical analyses.

As noted, we employed a geographic strategy of area coding based on present-day plate-tectonic boundaries, which are broadly congruent with modern zoogeographic regions (*e.g.*, Holt et al., 2013). This approach has distinct advantages and limitations. Chief among the benefits is its discreteness, objectivity, and unambiguousness, and it can be replicated easily in other taxa for comparative analyses. This eliminates subjective uncertainty regarding where to draw lines between terrestrial ecoregions like the Nearctic and Tropical Middle America (*e.g.*, Kreft & Jetz, 2010; Pyron, 2014a; Pyron, 2014b; Vilhena & Antonelli, 2015). Disadvantages include fixing boundaries between terrestrial ecoregions to their paleogeographic origins, which may not correspond with empirical evidence of the recent ecologically mediated biogeographic processes affecting related species in those regions (Mucina, 2018).

We attempt to avoid implying excessive certainty in our model-based reconstructions. While support for the most-likely ancestral range is high at nearly all internal nodes (Fig. S3; Table 2), we nevertheless concede that there are several factors that may confound such inferences. The first is the existence and phylogenetic placement of extralimital fossil taxa, species that are outside the modern range of the clade, which therefore does not reflect its historical distributions (Head, 2021). The second is topological uncertainty, such as the placement of Dibamia or relationships within Iguania (Burbrink et al., 2020). Third is variation in divergence-time estimates for nodes across the tree (see Jones et al., 2013; Simões & Pyron, 2021). While there are disparate estimations between phylogenomic (Irisarri et al., 2017) and total-evidence data (Pyron, 2017) especially for groups such as Gekkota, it is unlikely to affect the ancestral range estimation. Where differential divergence times can potentially impact results is in dispersal *versus* vicariance estimations, which is not something we attempted to capture in our study. Fourth are conceptual deficiencies in all existing biogeographic models, which include inadequate parameterization of extinction (see Matzke, 2014), and incomplete integration of correlated diversification and range evolution (see Goldberg, Lancaster & Ree, 2011; Goldberg & Igić, 2012; Caetano, O’Meara & Beaulieu, 2018). Recent results suggest that fully identifiable models for the latter may not be possible (see Louca & Pennell, 2020).

Regardless, we are confident that our results represent a substantial advance in presenting at least a partially informed view of the early biogeographic history of squamates, one that proposes several distinct hypotheses that can be tested further. Future studies could incorporate other parameters, such as trait-based dispersal (Klaus & Matzke, 2020), dispersal as a function of ecological or geographic distance between areas (Dam & Matzke, 2016). Body size (*e.g.*, Feldman et al., 2016), limb reduction (Vidal et al., 2008), and the temperate/tropical transition (Pyron, 2014b) all potentially impact dispersal and are avenues worth pursuing.

Finally, a recent methodology aimed at incorporating both fossil geographic occurrences and complex geographic interaction across time has been used for ancestral range estimations (Landis, 2017), and could be implemented in future studies. To do this effectively, more Jurassic squamate fossils need to be included in morphological matrices for a better understanding of their phylogenetic relationships (see Conrad, 2008; Wiens et al., 2010; Gauthier et al., 2012; Reeder et al., 2015; Pyron, 2017; Simoes et al., 2018). Paramacellodid lizards in particular will likely have a strong bearing on these estimates, given their Jurassic and early Cretaceous distribution across Pangaea and uncertain placement within Squamata (see Bittencourt et al., 2020). There is a well-known bias in the fossil record towards species in Laurasia (Vilhena & Smith, 2013; Valenzuela-Toro & Pyenson, 2019; Croft & Lorente, 2021), which may have impacted our results here, as all squamates available were from Laurasia in the Jurassic and early Cretaceous. In particular, the results presented here suggesting a Eurasian regionalization for Squamata would be falsified by fossil occurrence of relevant lineages in former Gondwanan continents such as South America, Australia, India, or Antarctica. Prospecting and research on these locations could greatly benefit future studies.

### Origin and diversification of extant Squamata

The phylogenetic relationships and present-day distributions of living squamates, along with well-constrained fossil taxa from the Jurassic and early Cretaceous, contain signal suggesting that the earliest squamate lineages (represented by the basal branches of the phylogeny of extant species) were localized along the plates that formed the coastline of the paleo-ocean, the Tethys Sea (Zhu, Zhao & Zhao, 2022; Fig. 3). Continuing into the Jurassic, a strongly supported pattern emerges of Eurasian origin for many major groups of squamates as Gondwana and Laurasia continued to break apart (Fig. 3). This geographical regionalization persisted at least until the mid to late Cretaceous, when Gondwanan radiations were established in Serpentes for Alethinophidia and Scolecophidia, the two major lineages of snakes (Fig. 3).

Later in the Cretaceous, Teiidae and later-diverging Gekkota also are reconstructed to have Gondwanan ancestral ranges (Fig. 3, for exact ranges see Appendix S2). These localizations formed *via* contraction from Pangaean or Northern Pangaean in Gekkota and Serpentes in contrast to Teiidae, which resulted from a jump dispersal from Laurasia to Gondwana (Fig. 3). This is coincident with and likely related to a substantial period of tectonic vicariance as the paleocontinents continued to diverge (Bird, 2003). It is worth noting that four of the nodes described above were constrained utilizing fossils and may seem circular. However, fossils are the only concrete evidence of the presence of a group in an area. Therefore, we argue that the estimated ancestral ranges are empirically valid and supported, since we are employing the increased certainty that comes along with fossil geographic occurrences as empirical data.

At more recent timescales, our results are congruent with most studies using molecular time trees to infer biogeographic histories to the extent that the area codings are comparable. For more detail, we refer to previous works on Dibamia (Townsend, Leavitt & Reeder, 2011), Gekkota (Agarwal et al., 2014; Carranza et al., 2000; Carranza & Arnold, 2006; Gamble et al., 2011; Gamble et al., 2008; Šmíd et al., 2013), Scincidae (Honda et al., 2000; Carranza & Arnold, 2003; Whiting et al., 2006; Brandley et al., 2011; Barley et al., 2015; Pereira & Schrago, 2017), Teiidae (Giugliano, Collevatti & Colli, 2007), Amphisbaenia (Longrich et al., 2015; Vidal et al., 2008), Lacertidae (Arnold, Arribas & Carranza, 2007; Tamar et al., 2016), Anguimorpha (Macey et al., 1999; Vidal et al., 2012), Iguania (Macey et al., 2000; Noonan & Sites Jr, 2010; Okajima & Kumazawa, 2010; Grismer et al., 2016), and in snakes, Typhlopoidea (Adalsteinsson et al., 2009; Vidal et al., 2010), Booidea (Graham Reynolds, Niemiller & Revell, 2014; Noonan & Chippindale, 2006a), and Colubroidea (Burbrink & Lawson, 2007; Alfaro et al., 2008; Wüster et al., 2008; Guo et al., 2012; Chen et al., 2013; Chen et al., 2017).

Subsequent studies may take a more focused perspective on individual lineages to enhance our understanding of the complex geographic history of Squamata. For example, colubroid snakes and Old World skinks have a vast, complex, and rather recent biogeographic history that deserves more attention than given here or in recent studies (see Cadle, 1985; Greer, 1970 for some early hypotheses and discussion). Similarly, little recent attention has been paid to teiid, lacertid, or anguimorph lizard biogeography at the continental or global scale since updated molecular phylogenies have become available (*i.e.,* Tonini et al., 2016).

A final question of great paleoecological and biogeographic interest is the directionality or asymmetry of dispersal between the landmasses analyzed here. Recent studies have shown that a variety of taxa experienced higher rates of dispersal into North America from Asia across Beringia than the opposite (the Cenozoic Beringian Dispersal Hypothesis; Guo et al., 2012; Jiang et al., 2019). Similarly interesting results might be obtained by examining rates of interchange between North and South America (see Estes & Báez, 1985 for early speculation), between Africa and western Eurasia (*e.g.*, Georgalis, Villa & Delfino, 2016; Tamar et al., 2016), between India and Eurasia (*e.g.*, Agarwal et al., 2014; Datta-Roy et al., 2012; Grismer et al., 2016), and between Australasia and eastern Eurasia (see Oliver & Sanders, 2009) during the Cenozoic. A great deal remains to be learned about the biogeographic history of Squamata, and our results will provide a robust foundation for productive future investigations.

Preliminary assessment of our Cenozoic results (Fig. 4A) reveals relatively few lineages (only 463 out of 5,415 species, <10%) with a recent Pangaean (*i.e.,* cosmopolitan) distribution. Regarding Cenozoic changes in distribution, there are very few range transitions from Laurasia to Gondwana (only 143 instances) and Gondwana to Laurasia (only 124 instances), suggesting a low rate of recent jump dispersal between the two former supercontinents. Accordingly, the network analysis (Fig. 4B) reveals a much clearer pattern of dispersal in the Cenozoic, which clearly shows a separation between clusters of dispersal within the New World (South America, North America, and the Caribbean) and Old World (Arabia, Australia, Africa, Eurasia, Sunda, and India), and very few events between the Old and New Worlds. This contrasts with the Gondwana-Laurasian division seen in our data during the Mesozoic (Fig. 3).

Additional fossil sampling, particularly in the Southern Hemisphere, will likely increase our knowledge of early Gondwanan squamate evolution. These data may broaden the ancestral range estimate to ‘Pangaea’ (see San Mauro et al., 2005 for a similar situation in Amphibia), completely alter it to ‘Laurasia’ or a component thereof (see (Zhang et al., 2013) for a similar result in plants), or a Gondwanan estimate with additional earlier dispersals into Laurasian landmasses (see (Gardner, Surya & Organ, 2019) for a similar example in early tetrapodomorphs). Recent Antarctic discoveries of an Eocene frog (Mörs, Reguero & Vasilyan, 2020) and late Cretaceous mosasaur material (Martin, 2006; Legendre et al., 2020) suggests the potential for a rich fossil history on this continent, which is almost entirely unknown in studies of most organisms (see Noonan & Chippindale, 2006a). Although Antarctica poses no significance for our study (as no modern squamates occupy the continent), other terrestrial tetrapod groups and marine squamates have been found in Antarctica (Goin & Goin, 1972; Rozadilla et al., 2016; Estrella et al., 2019), suggesting that with future exploration, potential terrestrial Antarctic squamates would expand our understanding of the biogeographic patterns on the continent.

## Conclusions

We find support for a Pangaean origin of early crown Squamata in the Jurassic followed by strong regionalization to Eurasia for subsequent Jurassic lineages, with little evidence for early occurrence in Australia, India, or Antarctica from phylogenetic or fossil evidence. The inclusion of well-constrained fossil areas supports a Eurasian component in estimated ancestral ranges. Subsequent regionalization and localization through range contraction resulted in Laurasian and Gondwanan endemism for the ancestral range of many extant groups by the end Cretaceous. Relatively simple Mesozoic patterns driven primarily by tectonic vicariance give way to complex Cenozoic histories reflecting a strong influence of long-distance dispersal. Preliminary evaluation of Cenozoic distribution patterns suggests frequent but potentially asymmetric transitions between and among Gondwanan/Laurasian and New World/Old World landmasses. More extensive inclusion of fossil taxa could dramatically impact the results in Squamata, as our reconstructions might otherwise seem to be at odds with the dearth of Jurassic fossil history present in Gondwana or early diverging squamates in Laurasian Europe (Simoes et al., 2018). These dynamics represent an intriguing source of future hypotheses across Squamata.

##  Supplemental Information

10.7717/peerj.17277/supp-1 Supplemental Information 1 Raw Data for Biogeographic Regions The first sheet contains the individual plate names and the major plate to which they were concatenated. The other sheets showed the raw data for each species and their regional assignments.

10.7717/peerj.17277/supp-2 Supplemental Information 2 The raw data of the results of the DEC+J results for the highest likelihood ancestral state reconstruction for the nine areas for each internal node

10.7717/peerj.17277/supp-3 Supplemental Information 3 R Script for BioGeoBEARS Along with Code for Nodal Constraints The complete code used to run BioGeoBEARS, in addition to the methods we used to constrain nodes to include fossil data in our analysis.

## Additional Information and Declarations

### Data Availability

The following information was supplied regarding data availability: The designated different biogeographic ranges and the code we used to run our tree and range file in BioGeoBEARS is available in the Supplementary Files.
