# Supplementary material for: Fossil-informed biogeographic analysis suggests Eurasian regionalization in crown Squamata during the early Jurassic
Source: PeerJ. 2024 Apr 30;12:e17277. doi: 10.7717/peerj.17277 (PMC11067913; doi:10.7717/peerj.17277)

BioGeoBEARS DEC+J on Squamata M0_unconstrained
ancstates: global optim, 4 areas max. d=0.0015; e=0; j=8e−04; LnL=−7297.26

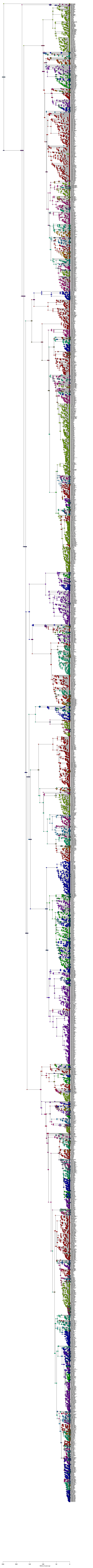

Supplement: Supplemental Information 2 [file peerj-12-17277-s002.pdf]
